# Digital Innovation and Integrated Care in People With Diabetes in Western Sydney: Retrospective Cohort Study

**DOI:** 10.2196/64832

**Published:** 2025-09-11

**Authors:** Ummul Mahfuza, Gideon Meyerowitz-Katz, Riham Abdulla Rasheed, Helen Dick, Glen Maberly, Rajini Jayaballa

**Affiliations:** 1 Western Sydney Diabetes New South Wales Australia; 2 Western Sydeny Diabetes University of Wollongong Wollongong Australia

**Keywords:** CGM, T2DM, digital innovation, integrated care, effectiveness, type 2 diabetes care, type 2 diabetes, Australia, diabetes, adoption, digital technologies, diabetes management, continuous glucose monitoring, glucose monitoring, diabetes specialist, diabetes educator, cohort, virtual consultations, daily insulin dose, insulin, disease management, patient engagement, primary care, chronic disease management

## Abstract

**Background:**

The COVID-19 pandemic catalyzed the adoption of digital technologies in health care. This study assesses a digital-first integrated care model for type 2 diabetes management in Western Sydney, using continuous glucose monitoring (CGM) and virtual Diabetes Case Conferences (DCC) involving the patient, general practitioner (GP), diabetes specialist, and diabetes educator at the same time.

**Objective:**

This study aims to assess the effectiveness of the innovative diabetes clinics in Western Sydney.

**Methods:**

In 2020, a total of 833 new patients with type 2 diabetes were seen at Western Sydney Diabetes (WSD) clinics. An early cohort of 103 patients was evaluated before and after participation in virtual DCC, incorporating CGM data analysis, digital educational resources, and remote consultations with a diabetes multidisciplinary team. Assessments were conducted at baseline and 3-4 months post DCC.

**Results:**

The integration of CGM and virtual consultations significantly improved glycemic control. Hemoglobin A_1c_ (HbA_1c_) levels decreased notably from 9.6% to 8.2% (average reduction of 1.4%; 95% CI 1.03-1.82; *P*<.001). Time in range (TIR) as measured by CGM increased substantially from 46% to 73% (95% CI 20-32; *P*<.001), and the glucose management indicator (GMI) improved from 7.9% to 7% (average reduction of 0.9%; 95% CI 0.55-1.2; *P*<.001). Despite no significant change in the total daily insulin dose, the proportion of patients on insulin therapy rose from 27% to 39% (*P*<.001), indicating more targeted and effective diabetes management.

**Conclusions:**

Our findings demonstrate the effectiveness of a digitally enabled integrated care model in managing type 2 diabetes. The use of CGM technology, complemented by virtual DCCs and digital educational tools, not only facilitated better disease management and patient engagement but also empowered primary care providers with advanced management capabilities. This digital approach addresses traditional barriers in diabetes care, highlighting the potential for scalable, technology-driven solutions in chronic disease management.

## Introduction

Diabetes presents a significant global health challenge. Rates of diabetes have increased dramatically in recent years, with disadvantaged regions bearing a greater burden when compared with wealthier areas of the world [1,2]. Western Sydney is a large geographic region in Sydney with 1 million residents. It is one of the most culturally diverse areas in Australia and has a number of suburbs with extremely disadvantaged populations. The rates of diabetes in Western Sydney are up to double those of higher-income areas in the city [1].

Western Sydney has an estimated 100,000 people with diabetes in the region. This equates to 13.1% of the adult population of 760,000 [2], with a total population of 1 million people in the region. This represents an unsustainable burden of disease that cannot be managed through existing specialist services that focus on treating individual patients. These traditional services, which usually require ongoing specialist care for every person diagnosed with diabetes, are too labor-intensive for the relatively small specialist workforce in the region. Recognizing this, Western Sydney Diabetes (WSD), an integrated care initiative located in the region, has pioneered a hybrid model of integrated care, designed to address the complex needs of patients with type 2 diabetes [3]. This model leverages digital technologies—including CGM, virtual multidisciplinary DCC, and a suite of digital educational resources—to enhance the coordination between primary and specialist care and empower general practitioners (GP) with the tools and knowledge to manage diabetes more effectively. The program was first modeled before the pandemic with local providers and has since been expanded to include a range of hybrid services that can be provided virtual or face to face.

Central to this program is the upskilling of the community workforce. WSD has been working with community medical staff since the program was started in 2014, with a focus on integrated care that crosses the specialist and GP boundaries. Previous evaluations have shown the benefits of involving GPs and other community health care staff in the care of people with diabetes and in better integrating these services with the hospital team [4].

In addition, WSD has adopted the innovative use of continuous glucose monitoring (CGM) in the program. CGM devices measure glucose tissue levels every minute and can be left on for 2 weeks at a time. This contrasts with traditional self-monitoring of blood glucose, whereby patients conduct finger-prick tests on their own blood several times a day. While initially controversial for type 2 diabetes, CGM has become a proven tool in treatment, with a range of research demonstrating the utility of this device for diabetes management [5,6]. By integrating real-time glucose data into patient care, WSD facilitates a comprehensive, collaborative treatment planning process. This process not only involves the patient and their GP but also draws on the expertise of dietitians, diabetes educators, and other clinicians.

**Figure 1:**
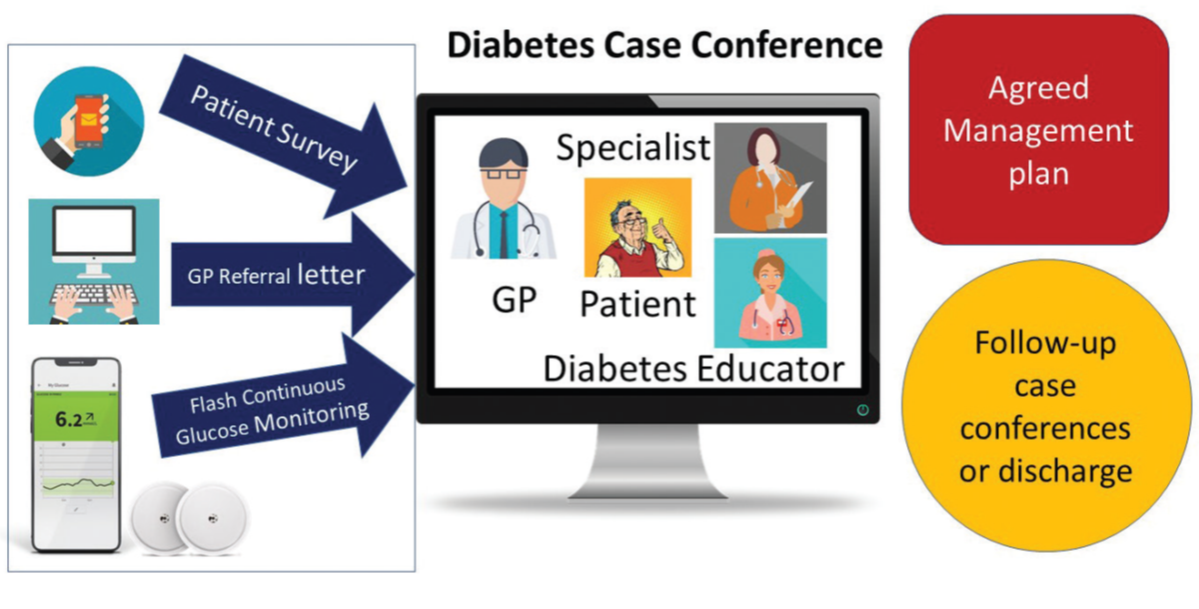
Western Sydney Diabetes model of care explained, patients referred by the general practitioner or other specialists are triaged, following triaging patients have preclinic work up including continuous glucose monitoring insertion before the case conference (adopted from Western Sydney Diabetes website).

WSD has also developed a comprehensive educational program for patients and providers. This includes diabetes case conferencing (DCC), which is a core component of the clinical care offered by WSD. DCC involves bringing together the patient, community providers, and hospital specialist team to discuss the patient’s case and make a plan for their treatment. This process aims to upskill the GP and team as well as provide gold-standard care for the patient. Previous evaluations have proven the benefit of this process not just for individual patients but for the GP’s practice of medicine as well [4,7]. The DCC model of care is depicted below in Figure 1.

WSD also conducts a yearly virtual diabetes masterclass series and in-person masterclass, which draws over 1000 registrants from across Australia. In addition, WSD has developed over 130 short educational videos for patients and providers to improve the understanding of diabetes [8]. These form a key component of the educational tools that WSD has developed to upskill practitioners in their work and help patients with self-management. All patients are provided with access to these digital tools when attending WSD clinics.

This combined clinical model, including a range of education and integration pieces, has not been formally evaluated. Previous studies have shown that individual aspects of this clinical model are effective, but existing data does not demonstrate any benefits to the model as a whole. Therefore, this study aims to retrospectively analyze the outcomes for patients managed under WSD’s innovative clinical model, focusing on the use of CGM and other technological solutions in regular practice for individuals with complex type 2 diabetes. By examining the model’s efficacy in improving patient care and enhancing GP capacity, the research seeks to provide valuable insights into the potential of integrated, technology-enabled care models to address the diabetes burden in high-prevalence regions.

## Methods

### Study Design and Participants

This retrospective cohort study analyzed patient records from the WSD complex diabetes clinics for the first quarter of 2022. Eligible patients had participated in at least one initial and one follow-up appointment at a WSD hybrid clinic, used CGM, and were subsequently discharged to their GP for ongoing diabetes management. Discharge criteria included the patient’s stability and the GP’s readiness to assume care, reflecting WSD’s objective to enhance GP capacity for diabetes management in the community.

### Data Collection

Data were extracted from the electronic medical records, focusing on clinical and demographic information, medication regimens, CGM data, and dietary interventions. Specifically, we extracted hemoglobin A_1c_ (HbA_1c_), total daily dose of insulin, and CGM metrics including time in range, glucose management indicator, and time above and below range. In addition, we collected detailed information about medication usage. A convenience sampling method was used, culminating in the collection of 103 patient records. Data were securely stored in a dedicated database for analysis.

We did not have specific inclusion or exclusion criteria beyond the sampling methodology. All patients with sufficient follow-up data who had been discharged by the clinic were included.

CGM data were gathered by clinical staff trained in CGM use and analysis. WSD exclusively used the Abbott Libre system during this period, and reports were extracted for all patients from the LibreView cloud system (Abbott Diabetes Care). A trained clinician then extracted the data from these reports and entered it into our research database.

### Statistical Analysis

Data analysis was conducted using Stata 15 (StataCorp). We used *t* tests and chi-square tests to compare pre- and postintervention clinical metrics for continuous and categorical variables, respectively. Key CGM metrics analyzed included time in range (TIR) and the glucose management indicator (GMI). Changes in medication, especially insulin dosages, were also examined.

### Qualitative Analysis

In addition to quantitative metrics, the study includes an informal qualitative review of CGM’s clinical utility in managing type 2 diabetes, assessing its impact on patient care and treatment outcomes. This qualitative review was conducted as a series of informal interviews with patients and GPs involved in the clinic. Themes from these discussions were collated and reported back by the WSD team.

### Ethical Considerations

This study was approved by the Western Sydney Local Health District Human Research Ethics Committee (approval 23/02578). The project was granted a waiver of consent as participants were deidentified before analysis.

## Results

### Patient Demographics and Baseline Characteristics

The study analyzed 103 patient records, with 81 patients having complete HbA_1c_ data both at baseline and follow-up. Table 1 provides a comprehensive overview of patient demographics. Patients were followed up between 3 to 6 months after their first attendance at the WSD clinic.

**Table 1:** Demographics and diabetes medications at baseline for the sample.

Variables		Male (n=61)	Female (n=42)	Total (N=103)
Age (years), mean (SD)		65.1 (10)	61.1 (13.1)	63.4 (11.5)
**Diabetes medications at baseline, n (%)**				
	Metformin	42 (68.9)	34 (81)	76 (73.8)
	Sulfonylurea	24 (39.3)	4 (9.5)	28 (27.2)
	DPP4I^a^	27 (44.3)	11 (26.2)	38 (36.9)
	SGLT2I^b^	33 (54.1)	12 (28.6)	45 (43.7)
	GLP1RA^c^	13 (21.3)	13 (31)	26 (25.2)
	Insulin	33 (54.1)	29 (69)	62 (60.2)
Mean insulin TDD^d^ in units, mean (SD)		33.6 (42.5)	39.9 (41)	36.2 (41.8)
Mean baseline HbA_1c_^e^ in %, mean (SD)		9.5 (1.5)	9.9 (1.7)	9.7 (1.6)

^a^DPP4I: Dipeptidyl Peptidase-4 Inhibitor.

^b^SGLT2I: Sodium-Glucose Cotransporter-2 Inhibitor.

^c^GLP1RA: Glucagon-Like Peptide-1 Receptor Agonist.

^d^TDD: total daily dose.

^e^HbA_1c_: Hemoglobin A_1c_.

### Glycemic Control

A significant reduction in HbA_1c_ levels was observed, decreasing from an average of 9.6% at baseline to 8.2% at follow-up. This change represents a mean reduction of 1.4% (95% CI 1.03-1.82%; *P*<001).

### CGM Metrics

CGM data highlighted an improvement in TIR, which increased from 46% at baseline to 73% at the follow-up (95% CI 20%-32%; *P*<001). In addition, the GMI decreased from 7.97% to 6.94%, a reduction of 1.03% (95% CI 0.55%-1.2%; *P*<001), indicating enhanced glucose control. Time above range (TAR) high and TAR-very high reduced by 10.06% and 16.95%, respectively. Time below range (TBR)-low and TBR-very low also reduced by 0.1% and 0.44%, respectively (Table 2).

**Table 2:** Changes in continuous glucose monitoring metrics comparing the initial review and before discharge.

CGM^a^ metrics	Initial review, mean (SD)	Before discharge, mean (SD)	Change	*P* value
Sensor active time (%)	75.44 (20.84)	78.61 (20.39)	3.17	.84
Glucose variability (%)	28.94 (7.85)	27.34 (5.92)	–1.6	.16
Glucose Management Indicator (GMI; %)	7.97 (1.31)	6.94 (0.71)	–1.03	<.001
Average blood glucose (mmol/L)	10.74 (3.20)	8.5 (1.51)	–2.24	<.001
Time in range (TIR) 3.9-10 mmol/L (%)	46 (27.69)	73 (15.97)	27	<.001
Time above range (TAR) – high 10.1-13.9 mmol/L (%)	31.32 (14.88)	21.26 (10.90)	–10.06	<.001
Time above range (TAR) – very high>14 mmol/L (%)	20.98 (22.22)	4.03 (8.25)	–16.95	<.001
Time below range (TBR) – low 3.1-3.8 mmo/L (%)	1.29 (2.63)	1.19 (2.55)	–0.1	.98
Time below range (TBR) – very low <3.0 mmo/L (%)	0.6 (1.83)	0.16 (0.93)	–0.44	.07

^a^CGM: continuous glucose monitoring.

### Medication Adjustments

Substantial changes in medication regimens were noted post consultation. The use of sulfonylureas decreased significantly, with only 17% (5/28) of patients continuing this medication at follow-up. Conversely, 32 patients had a glucagon-like peptide-1 receptor agonist (GLP1 RA) added to their regimen, while 9 discontinued its use. The proportion of patients on insulin therapy increased from 60% to 73%, although the average total daily insulin dose did not show a statistically significant change (average decrease of 5 units, *P*=.10).

### Patient and GP Experiences

Both patients and GPs expressed highly positive feedback regarding their experiences with the virtual clinic model. The use of CGM was particularly highlighted as beneficial. Patients reported improved understanding and management of their condition, attributing this to the insights gained from real-time glucose monitoring. GPs noted the virtual clinic facilitated more effective communication, and collaborative care planning, and enhanced their ability to manage diabetes in a primary care setting.

## Discussion

### Principal Findings

This study provided evidence of improvements in patient outcomes associated with the WSD model of care. This included improvements in CGM parameters, medication usage, and other key diabetes markers. While the study was limited, lacking a control group, the findings suggest that this model of care may be beneficial for similar clinical areas in the future.

The integration of CGM and virtual DCC within the WSD care model has marked a paradigm shift in managing complex type 2 diabetes. This innovative approach, blending digital health technologies with a collaborative care framework, has led to remarkable glycemic improvements, significantly surpassing outcomes commonly associated with telehealth interventions alone [9,10]. The essence of this success lies in the holistic care model, with CGM acting as a linchpin for real-time glycemic insight, thereby revolutionizing both self-management practices and clinical decision-making. The feedback in our informal review underscores the value of integrating digital health technologies in chronic disease management, aligning with the study's aim to evaluate the impact of a technologically enabled care model on diabetes management.

CGM has emerged as a critical tool in diabetes care, functioning as a “truth detector” by providing detailed insights into glucose fluctuations directly linked to lifestyle choices [11]. This real-time data empowers patients to observe the immediate impact of their dietary and exercise habits, facilitating more informed lifestyle adjustments and significantly improving glycemic control. However, CGM’s role in enabling efficient remote monitoring underscores its indispensability in our virtual care framework, aligning with the efficacy of telehealth in diabetes management demonstrated by previous studies [9].

Ehrhard NM [12] in a 52-week randomized controlled trial (RCT) showed HbA_1c_ reduced by 1% at 12 weeks with CGM compared with 0.5% with self-monitoring of blood glucose (SMBG). Furthermore, Vigersky et al [13] showed that after 12 weeks of intervention using CGM or SMBG, HbA_1c_ reduced by 0.8% in the CGM group and 0.2% in the SMBG group at 52 weeks. A recent systematic review and meta-analysis of 12 RCTs comprising 1248 participants by Jancev et al [14] showed CGM use resulted in an improvement of 6.36% for TIR and a 0.31% reduction in HbA_1c_.

The DCC component has been crucial in eliminating geographical barriers and fostering seamless collaboration between primary care providers and specialists. GPs can attend the appointments virtually, with patients joining either from their own homes, the GP clinic, or the specialist clinic. Administration support has been crucial to allowing this hybrid model to be successfully implemented [7]. This model not only guarantees continuity of care but also serves as an invaluable educational platform for GPs, bolstering their ability to manage complex type 2 diabetes cases more effectively. The educational aspect of DCC, alongside the practical insights gained from CGM data, illustrates the power of this model to scale diabetes management efforts, catering to a wider patient demographic in the evolving landscape of digital health care.

The Australian National Diabetes Strategy (2016-2020) [15] addressed key areas such as integrated care fostering shared care and a patient-centered approach. Several services, such as the Hunter New England diabetes service, which covers a very large geographic region roughly the size of Greece but with a population of just under 1 million, and WSD were highlighted in this strategy as exemplars of diabetes management and engagement with community [16]. Similar to WSD’s evaluation, the Hunter New DAP [17] case conferencing study showed improvement in HbA_1c_ by 0.4%. Another similar model in the southwest of Sydney demonstrated equivalent benefits in their patient cohort [18].

Another RCT demonstrated benefits for video case conferencing similar to those seen in this study [14]. WSD’s previous evaluations have also shown that with DCC patients were more empowered in shared decision-making and perceived as receiving good ongoing care further enhanced by the digital education bundles [15]. Table 3 shows the improvements associated with video conferencing for various studies on type 2 diabetes.

**Table 3:** Related researches show improvement in hemoglobin A1c (HbA1c) and other variables using virtual health in type 2 diabetes mellitus.

Study	HbA_1c_ improvement (%)	Blood pressure (mm Hg)	BMI (kg/m^2^)	Study size, n
Abrahamian et al [19]	0.3%	156→148 88→83	Nil	154
Basudev et al [20]	0.6 (1.7%) Control group 0.8 (1.9%)	6 (16) systolic 2 (18) control group	Nil	208
Zhou et al [21]	–2.4% (P <.001) Control -0.62	3 Control 2	—^a^	108
Sood et al [22]	–1.01% Control -0.68% P=.19	—	—	182
De Groot et al [23]	–0.486% P<.001	-0.875 P<.01	—	8410

^a^Not applicable

Like these previous studies, our investigation supports the use of DCC and CGM in the treatment of diabetes. While the study design does not allow for inferences about specific modalities of care, the integrated nature of the clinic is clearly important to improve the health of people with diabetes. In our previous clinical evaluations, we have shown an improvement associated with DCC of 0.87% in terms of HbA_1c_. That the improvement in this study is higher demonstrates the importance of innovative models of care and incorporating CGM into this process [4].

This care model is aimed at collaborating with community providers to improve the connection between specialist and community health services, and to upskill community health care workers to improve diabetes management more broadly. Our informal qualitative review indicated that this was occurring, with these findings supported by previous work, which has demonstrated the benefits more fully.

This study had many limitations. The inclusion criteria mean that only patients who improved were included in the cohort by definition. Therefore, it is not possible to draw concrete inferences about the improvements seen in this study and the measures used. There is no control group, and therefore there is no ability to draw causal inferences. The qualitative review was informal and lacked sufficient rigor to make clear statements about clinician or patient insights into the clinic. The sample size was relatively large, and the measurements were all objective; however, the retrospective nature of the study also limits the conclusions that can be drawn.

The study’s limitations highlight the necessity for broader, comparative research to affirm these findings across varied health care contexts. The considerable baseline HbA_1c_ levels also suggest that the dramatic improvements observed may not be universally achievable, indicating a need for tailored interventions based on patient baseline characteristics. There were also issues with the deployment of these interventions, highlighting the need for additional administrative support when implementing a complex technological program such as this.

### Conclusion

In conclusion, the WSD model shows that collaborative programs are implementable even in high-risk complex clinics. This integrative model is likely to have benefits more broadly for people with diabetes and community health care providers. Embracing and refining such innovative care approaches holds the promise of scaling diabetes management capabilities, potentially improving health outcomes for a broader spectrum of patients with type 2 diabetes. As we move forward, our focus will also extend to objectively measuring patient health outcomes in relation to engagement levels and social determinants, aiming to further enhance the care model’s efficacy and reach.

## Abbreviations

CGM: continuous glucose monitoring
DCC: diabetes case conferencing
GLP-1 RA: glucagon-like peptide-1 receptor agonist
GMI: glucose management indicator
GP: general practitioner
HbA1c: hemoglobin A1c
RCT: randomized controlled trial
SMBG: self-monitoring of blood glucose
TAR: time above range
TBR: time below range
TIR: time in range
WSD: Western Sydney Diabetes
